# Proteomics in India: the clinical aspect

**DOI:** 10.1186/s12014-016-9122-0

**Published:** 2016-11-05

**Authors:** Somaditya Mukherjee, Arun Bandyopadhyay

**Affiliations:** Cell Biology and Physiology Division, CSIR-Indian Institute of Chemical Biology, 4, Raja S. C. Mullick Road, Kolkata, 700032 India

**Keywords:** Clinical proteomics, Indian academia, Biological research, Translation

## Abstract

Proteomics has emerged as a highly promising bioanalytical technique in various aspects of applied biological research. In Indian academia, proteomics research has grown remarkably over the last decade. It is being extensively used for both basic as well as translation research in the areas of infectious and immune disorders, reproductive disorders, cardiovascular diseases, diabetes, eye disorders, human cancers and hematological disorders. Recently, some seminal works on clinical proteomics have been reported from several laboratories across India. This review aims to shed light on the increasing use of proteomics in India in a variety of biological conditions. It also highlights that India has the expertise and infrastructure needed for pursuing proteomics research in the country and to participate in global initiatives. Research in clinical proteomics is gradually picking up pace in India and its future seems very bright.

## Background

The beginning of the twentyfirst century, saw the birth of a new technology called ‘proteomics’ that emerged as a highly promising bioanalytical technique in various aspects of applied biological research. Though India could not play a role in human genome sequencing projects, the country has emerged as one of the frontrunners in global proteomics research and has come a long way from where it was, probably a decade ago [1–3]. The importance of proteomics research was realized when many Indian researchers initiated identifying proteins critical to the pathophysiology of various diseases. However, the actual momentum gained subsequently when several laboratories were successful in arranging sophisticated facilities for efficient proteome separation and detection using tissue as well as liquid samples. This was supported further with national efforts for network building and collaboration amongst various laboratories with a primary objective for early detection, diagnosis, and a way to therapeutics for common as well rare diseases.

Although the development of proteomics research in India was rather slow at the outset, the last decade or so has seen a dramatic expansion in the proteomics community [4]. Presently, there are over a hundred research laboratories in approximately 80 academic or research institutes across India involved in various proteome-level investigations. Thus it may safely be said that in the past few years, proteomics has emerged as a powerful tool for varied biological investigations in the country. Proteomics is also becoming an attractive method of choice for the identification and development of new biomarkers and potential therapeutic targets. Increasing financial and infrastructure support from the government is likely to take proteomics research in the country to new heights.

**Fig. 1:**
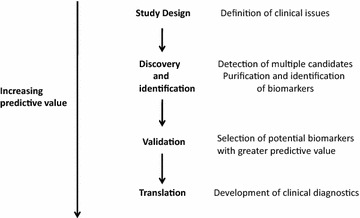
Clinical proteomics study design

Proteomics is a scientific approach that attempts to completely characterize the proteome (or subproteome) of a cell or tissue. Various technologies can be employed during the three major steps involved in proteomic analysis: sample preparation, separation of proteins or peptides, identification and characterization of proteins, and the technologies can be mixed and matched to meet the needs to answer any particular biological or clinical question. The advancement of proteomic technologies now addresses the challenges associated with the pathophysiology of various diseases. Proteins are most widely affected in disease, response and recovery. The most important application of proteomics is believed to be discovery of disease biomarkers and drug targets which can lead to designing of products aimed at diagnosis and treatment of diseases like cancer, cardiovascular diseases, obesity and type 2 diabetes. This application of proteomic tools and knowhow to the field of medicine is called clinical proteomics (Fig. 1). It encompasses the translation of proteomic detection technologies and strategies towards the production of diagnostics and therapeutics for the direct improvement of human health [5]. Therefore, clinical proteomics methods hold special promise for the identification of new biomarkers that might improve disease staging, risk stratification, and the prognosis and treatment of diseases. The following review intends to look into the current developments and shortcomings in India’s clinical proteomics scene and the problems faced by the Indian scientific community in translational research. The review also deals with next generation proteomics methods and different proteomics databases developed in India.

## Review

### Proteomics in Indian academia and the emergence of Proteomics Society India

Proteomics is taking centre stage in biology and biomedical research in India. Several institutes are currently employing proteomics techniques in their research and at present, we have an enviable mass of proteomics scientists in the country. To foster entrepreneurship and skill development in proteomics research, many biotech parks have been established with public and private support. In recent years, proteomics and related disciplines have also been incorporated into academic curricula across India due to their increasing impact on clinical research. For instance, The Clinical Proteomics Remote Triggering Virtual Laboratory (http://iitb.vlab.co.in/?sub=41&brch=237) creates a virtual platform for beginners to acquire knowledge and a realistic experience of performing different techniques commonly applied in clinical proteomics research. These new e-learning resources in proteomics act as important global platforms for students, e-learners and researchers. With expansion of research efforts and introduction of modern technologies, it is important to facilitate interactions among the proteomics fraternity and help them disseminate knowledge for the holistic development of this discipline. “Proteomics Society India”, is thus a step in that direction that provides a platform to pursue an agenda that will meet this objective. The Society which is deeply involved towards imparting education in proteomics research through organizing periodic conferences, workshops and meetings, is still in its infancy. However, a lot is being done to make students and teachers familiar with proteomics. Consequently, the 3rd Annual Meeting of PSI—International Proteomics Conference on ‘OMICS MEETS DISEASE’, was held at Kolkata in December 2011. This was followed by a themed conference on ‘Medical Proteomics’ in Bangalore in November,2013. Both the events were well attended by clinicians, eminent scientists and students. This highlights India’s emergence as a global player in cutting-edge clinical proteomics research.

### Methods adopted for clinical proteomics research in India

In India, clinical proteomics efforts initially relied upon the traditional 2DE-MS approach and its advanced variation, 2D-DIGE. However, these gel-based protein separation approaches offered limited proteome coverage. This resulted in a shift towards adopting alternative, high throughput shotgun approaches such as liquid chromatography–mass spectrometry (LC–MS or LC–MS/MS). This approach, having greater quantitative ability, enabled deeper proteome analysis generating huge protein datasets. This gave way to semi quantitative labeling technologies such as isobaric tags for relative and absolute quantitation (iTRAQ) and stable isotope labeling by amino acids in cell culture (SILAC). iTRAQ is a mass spectrometry based quantitative proteomics method that determines the amount of proteins from different sources in a single experiment using stable isotope labeled molecules that can be covalent bonded to the N-terminus and side chain amines of proteins [6–8]. Candidate tissue, serum and drug resistance biomarkers have been discovered using this technique. SILAC is another mass spectrometry based quantitative proteomic technique that detects differences in protein abundance among samples using non-radioactive isotopic labeling [9–12]. It has emerged as a very powerful method to study cell signaling, post-translational modifications such as phosphorylation, protein–protein interaction and regulation of gene expression [13, 14]. SILAC has become particularly important in the field of secretomics.

In general, mass spectrometry-a central tool used for monitoring, identifying and characterizing proteins has become an invaluable technique of choice for both protein profiling as well as for positive protein identification. With an increasing ability to correctly characterize reduced quantities of samples and more complex mixtures of proteins and peptides, mass spectrometry is quickly becoming a key tool in the discovery of alterations to the proteome thereby facilitating biomarker identification. Additionally, the possibility of using mass spectrometry directly in clinical assays is also becoming a reality because the technique has a low detection limit and can perform high throughput analyses. Though scientists prefer the antibody based targeted approaches (ELISAs, TMAs) for confirmation and validation of markers, targeted quantitative approaches like MRM are becoming quite popular in the proteomics community. MRM is highly reproducible and is able to replace expensive antibody-based quantification like Western blotting and ELISA. Many independent investigations have used this approach for targeted quantification. One such example is quantification of cancer related proteins in body fluids using targeted proteomics [15]. Multiple reaction monitoring (MRM) using mass spectrometry is a highly sensitive and selective method for the targeted quantitation of protein/peptide abundances in complex biological samples [16]. Parallel reaction monitoring (PRM) detects all product ions and identifies peptides with high confidence. Thus, interference is reduced and specificity is increased in PRM. Qquantification in PRM is done by extracting one or more fragment ions that are selected post-acquisition. However, data visualization and analysis in PRM is similar to MRM [17]. Compared to MRM, PRM provides data with better mass accuracy and removes noise of interfering signals. In addition, PRM based targeted proteomics reduces assay development time compared to MRM because in PRM, fragment ions can be selected post-acquisition. The high scan speed facilitates development of sequential window acquisition of all theoretical mass spectra (SWATH). Peptides are quantitated by targeted extraction of SWATH-MS data. However, direct proteome profiling has remained a major technical challenge for body fluid proteomics particularly serum and plasma. Hence, it may be concluded that conventional tools for doing proteomics research such as 2DE to separate a large number of proteins, spot picking, in-gel trypsin digestion of excised gel spots and MALDI–TOF–MS based identification are gradually giving way to more advanced proteomics tools.

Recent developments in next generation proteomics tools are yielding newer information in modern biology. MALDI imaging mass spectrometry (MALDI–IMS**)** is providing major contributions to the understanding of disease progression, improving diagnostics, and drug delivery. MALDI–IMS is a mass spectrometry based imaging technique which allows direct label free measurement of biomolecules, metabolites and drugs at one time in tissue sections without destroying the sample [18, 19]. Proteogenomics refers to use of proteomic information, generated from mass spectrometry, to improve gene annotations [20–22]. Proteogenomic studies can also provide information about the presence of programmed frameshifts, N-terminal methionine excision, signal peptides, proteolysis and other post-translational modifications [21]. Proteogenomics is becoming increasingly relevant in clinical research and biomarker discovery. Protein microarray is a sensitive and high throughput gel-free protein analysis method that is rapidly becoming powerful for protein detection, protein expression profiling, diagnostics, biomarker discovery, protein–protein interactions etc. These arrays are solid-phase ligand-binding assay systems using immobilized proteins of interest on surfaces which include membranes, glass, microtiter wells, mass spectrometer plates, beads etc. [23].

### Studies in clinical proteomics carried out so far in India

As in other parts of the world, proteomics has been implemented by Indian researchers to elucidate biological mechanisms as well as to discover biomarkers for various diseases, enabled by advancements both in instrumentation and software. Considerable efforts have been made to explore the mechanism of disease pathogenesis using animal models as well as cell biology and molecular biology techniques. The publications from these studies have emerged from major research institutes and University laboratories from across the country. Several research groups from India are actively pursuing cutting-edge research on proteomics of different infectious and immune disorders, reproductive disorders, cardiovascular diseases, diabetes, eye disorders, human cancers and hematological disorders.

#### Infectious disease and immune disorders

Proteomics is employed as a study tool in tuberculosis which affects a large population in the country and is of major concern. Research institutes like National JALMA Institute for Leprosy & Other Mycobacterial Diseases, Agra and National Institute for Research in Tuberculosis, Chennai are the frontrunners in this area. The underlying objective is to characterize the various strains, decoding drug resistance and identifying biomarker candidates along with new therapeutic targets [24, 25]. Proteomics of clinical isolates of malarial parasites is undertaken by institutes such as IISc, Bangalore; ICGEB, New Delhi and IIT Powai, Mumbai. These efforts are providing insights into its physiology whereas proteomic analysis of serum from infected individuals gives preliminary information concerning potential biomarkers [26–29]. Several studies have also been conducted on clinical isolates of wild type and drug resistant varieties of Leishmania across different institutes of the country like NCCS, Pune; JNU, New Delhi; CDRI, Lucknow; IOB, Bangalore; AIIMS, New Delhi; NIPER, Mohali; BHU, Varanasi and IICB, Kolkata [30–44]. Interesting findings have also been reported on other infectious diseases like hepatitis, food poisoning, leprosy etc. [45–50]. Substantial research activities on some other immune disorders have also been carried out in centres like Bose Institute, Kolkata; PGIMER, Chandigarh; IOB, Bangalore; CDRI, Lucknow; IGIB, New Delhi and AIIMS, New Delhi using high throughput proteomic techniques [51–60].

#### Reproductive biology and respiratory disorder

Proteomic analysis of germ cells, seminal plasma and mammary epithelial cells is carried out at NDRI, Karnal to know about fertility and lactation in dairy animals [61–63]. Proteomics studies on reproductive disorders such as endometriosis and polycystic ovarian syndrome have also been reported from institutes like IIT Kharagpur and NIRRH, Mumbai [64–66]. The effect of hypobaric hypoxia occurring at high altitude is also being analyzed by a research group at DIPAS, DRDO, New Delhi through proteomics methods [67].

#### Metabolic diseases

Proteomic technologies have also been used to understand cardiovascular diseases like Cardiac Hypertrophy, CAD, MI, and RHD. Several renowned institutes like PGIMER, Chandigarh; IGIB, New Delhi; IICT, Hyderabad; Thrombosis Research Institute, Bangalore and IICB, Kolkata are involved in such initiatives [68–75]. The difference between stenotic and non stenotic human plasma proteins has been investigated in the context of rheumatic mitral valve disease which is prevalent in developing countries including India. Proteins, which manifest both inflammatory and thrombotic components have been identified in Rheumatic Mitral Stenosis at IICB, Kolkata using a label free proteomic approach, [75]. Very recently, iTRAQ based proteomic studies from IGIB, New Delhi have shown that downregulation of apolipoproteins and albumin might be responsible for impaired reverse cholesterol transport in stable CAD [76]. Interesting findings in Type 2 diabetes have emerged from proteomic studies carried out at NCL, Pune in human plasma and drug induced animal models [77–85]. One of the novel initiatives undertaken by this group is to study advanced glycation end products (AGE) in a diabetic animal model using MALDI–TOF–MS based proteomics [82].

#### Eye disease

Proteomics research on various eye disorders like diabetic retinopathy, mycotic keratitis, dry eye syndrome and retinoblastoma tumors have been undertaken across institutes like SRM University, Kattankulathur; Aravind Medical Research Foundation, Aravind Eye Care System, Madurai and Sankara Nethralaya, Chennai [86–92].

#### Cancers and hematological disorders

The above studies help us to understand that proteomics is now universally applied to study various types of circulating body fluids and other biological samples. For instance, proteome profiles of serum/plasma, other biological fluids and biopsy specimens from different types of cancers are currently being evaluated across centres like IISc, Bangalore; CCMB, Hyderabad; IOB, Bangalore; ACTREC, Mumbai and IIT Powai, Mumbai with necessary infrastructural support, scientific knowledge and technical expertise from the institutes as required [93–109]. Similar studies have been replicated for different hematological disorders at SINP, Kolkata [110–113]. These examples give an idea of some of the areas where proteomic tools are being currently used.

Newer separation methods for pre-fractionation and histochemical quantification of stained tissue sections are gaining popularity by the day [114–123]. Exosomes, are also becoming an attractive starting material for the analysis of circulatory biomarkers. The impact of these studies on basic as well as biomedical research will only be felt in the years to come.

### Different proteomics databases developed in India

With the expansion of protein sequence databases and search engines designed for direct input of mass spectrometry data, the high-resolution mass spectrometers with greater sensitivities can provide rapid and accurate identification of proteins for any number of species for which complete or partial genomes have been sequenced. This has lured scientists and data analysts to use proteomic methods in biomedical investigations and generate vast amounts of biological and molecular data that are invaluable to the global proteomics community. Such diverse datasets help to identify enzyme-substrate, signaling and coordination networks involved in various biological processes, which in turn, can be helpful in molecular diagnostics and disease therapeutics. Progress in the field of proteomics will benefit substantially from the development of informatics programs which enable faster processing of complex datasets. Thus, bioinformatics is one of the pillars of proteomics. Researchers at IOB (http://ibioinformatics.org), Bangalore have developed sophisticated bioinformatics tools like HPRD (www.hprd.org), Human Proteinpedia (www.humanproteinpedia.org/) and Human Proteome Map, a database on pancreatic cancers and several human signaling pathways [124–126]. It is through creation of the ‘Human Proteome Map’ that India received global attention recently. Using high-resolution mass spectrometry, independent drafts of the ‘Human Proteome Maps’ have been published by two investigator led groups which only highlights the biological significance of the data [126]. NetPath (http://www.netpath.org)—the flagship database of IOB, contains manually curated data for 36 human signaling pathways [127–134]. Signaling pathways such as Delta-Notch, EGFR1, Hedgehog, TNF-alpha have been curated at IOB, Bangalore. Particularly, the Wnt signaling pathway has been selected and organized for Cancer Cell Map (http://cancer.cellmap.org/cellmap/), which is a database of pathways focusing on human cancer. NetSlim is a slimmer collective version of NetPath (http://www.netpath.org/netslim/). It contains a graphical network of major signaling reactions [135]. Research teams at Lab SurgPath, Mumbai have contributed significantly to the Human Protein Atlas (http://www.proteinatlas.org/).

At the IMTECH, Chandigarh a curated database of proteins associated with cervix cancer—CCDB, a database of anticancer peptides and proteins called CancerPPD, and a database of hemolytic and non-hemolytic peptides—Hemolytik have been developed [136, 137]. NCBS has also come up with the DOQCS, which represents the collection of basic models of different signaling pathways [138–141]. Researchers at IISc, Bangalore have developed quite a few protein databases related to structure and function of protein kinases like ‘KinG’, NrichD, PALI, PRODOC and MulPSSM [142–144]. A number of protein databases such as PepBind, Immune Epitope Prediction Database & Tools, SEDB, Clostridium-DT(DB), and VPDB have originated from the Center of Bioinformatics in Pondicherry University [145–147]. Databases of protein features, resources for signaling and metabolic pathways intrinsic to specific biological processes, and repositories of disease-specific molecular level changes will help researchers use systems biology approaches to understand disease mechanisms in greater detail.

## Conclusion

Clinical proteomics is yielding some fruitful results for Indian proteomics researchers utilizing state-of-the-art experimental and analytical methodologies. All these will benefit the proteomics community in the long run. However, these efforts are not enough for the benefit of the huge Indian patient population. Despite some success stories, India is still some distance away from successful translation of laboratory findings into clinical practice. India needs focused, sustained policies to promote translational research through specialized mega projects. Establishment of tissue repositories or registries is the need of the hour. The country’s overall healthcare infrastructure and medical informatics system also needs both public and private sector support on a long term basis so as to facilitate more effective translation studies. Tie-ups with commercial companies to facilitate technological developments will be necessary. At present, most of the proteomic investigations are arising from individual labs in research institutes of national repute. Almost all the studies are in the discovery phase. Collaboration between scientists and clinicians will facilitate the execution of well-designed multi-institutional and multi-centric studies aimed at addressing the relevant health problems unique to India, evaluating disease outcomes and their validity.

## Abbreviations

2DE: two–dimensional electrophoresis
2D DIGE: two-dimensional difference gel electrophoresis
ACTREC: Advanced Centre for Treatment, Research and Education in Cancer
AIIMS: All India Institute of Medical Sciences
BHU: Banaras Hindu University
CAD: coronary artery disease
CCMB: Centre for Cellular and Molecular Biology
CDRI: Central Drug Research Institute
DIPAS: Defence Institute of Physiology & Allied Sciences
DOQCS: Database of Quantitative Cellular Signaling
DRDO: Defence Research and Development Organization
EGFR1: epidermal growth factor receptor 1
ELISAs: enzyme linked immunosorbent assays
HPRD: Human Protein Reference Database
ICGEB: International Centre for Genetic Engineering and Biotechnology
IGIB: Institute of Genomics and Integrative Biology
IICB: Indian Institute of Chemical Biology
IICT: Indian Institute of Chemical Technology
IISc: Indian Institute of Science
IIT: Indian Institute of Technology
IMTECH: Institute of Microbial TECHnology
IOB: Institute of Bioinformatics
LC–MS: liquid chromatography–mass spectrometry
LC–MS/MS: liquid chromatography–tandem mass spectrometry
MALDI-TOF MS: matrix assisted laser desorption ionization-time of flight mass spectrometry
MI: myocardial infarction
MRM: multiple reaction monitoring
NCBS: National Centre for Biological Sciences
NCCS: National Centre for Cell Science
NCL: National Chemical Laboratory
NIPER: National Institute of Pharmaceutical Education and Research
NIRRH: National Institute for Research in Reproductive Health
PepBind: Peptide Binding Protein Database
PGIMER: Postgraduate Institute of Medical Education and Research
R&D: Research and Development
SEDB: Structural Epitope Database
SINP: Saha Institute of Nuclear Physics
TMAs: tissue microarrays
VPDB: Viral Protein Structural Database
